# Organotypic Hippocampal Slice Cultures from Adult Tauopathy Mice and Theragnostic Evaluation of Nanomaterial Phospho-TAU Antibody-Conjugates

**DOI:** 10.3390/cells12101422

**Published:** 2023-05-18

**Authors:** Susanna Kemppainen, Nadine Huber, Roosa-Maria Willman, Ana Zamora, Petra Mäkinen, Henna Martiskainen, Mari Takalo, Annakaisa Haapasalo, Tomás Sobrino, Manuel Antonio González Gómez, Yolanda Piñeiro, José Rivas, Uwe Himmelreich, Mikko Hiltunen

**Affiliations:** 1 Institute of Biomedicine, University of Eastern Finland, 70211 Kuopio, Finlandmikko.hiltunen@uef.fi (M.H.); 2 A.I. Virtanen Institute for Molecular Sciences, University of Eastern Finland, 70211 Kuopio, Finland; 3 Molecular Imaging and Photonics, KU Leuven, 3001 Leuven, Belgium; 4 Biomedical MRI, Department of Imaging and Pathology, KU Leuven, 3000 Leuven, Belgium; 5 NeuroAging Group (NEURAL), Clinical Neurosciences Research Laboratory (LINC), Health Research Institute of Santiago de Compostela (IDIS), 15706 Santiago de Compostela, Spain; 6 Institute of Materials, Applied Physics Department, Universidade de Santiago de Compostela, 15782 Santiago de Compostela, Spain

**Keywords:** organotypic slice culture, adult tissue, hyperphosphorylated TAU, P301S mice, ex vivo, theragnostic nanomaterials, immunotherapy

## Abstract

Organotypic slice culture models surpass conventional in vitro methods in many aspects. They retain all tissue-resident cell types and tissue hierarchy. For studying multifactorial neurodegenerative diseases such as tauopathies, it is crucial to maintain cellular crosstalk in an accessible model system. Organotypic slice cultures from postnatal tissue are an established research tool, but adult tissue-originating systems are missing, yet necessary, as young tissue-originating systems cannot fully model adult or senescent brains. To establish an adult-originating slice culture system for tauopathy studies, we made hippocampal slice cultures from transgenic 5-month-old hTau.P301S mice. In addition to the comprehensive characterization, we set out to test a novel antibody for hyperphosphorylated TAU (pTAU, B6), with and without a nanomaterial conjugate. Adult hippocampal slices retained intact hippocampal layers, astrocytes, and functional microglia during culturing. The P301S-slice neurons expressed pTAU throughout the granular cell layer and secreted pTAU to the culture medium, whereas the wildtype slices did not. Additionally, cytotoxicity and inflammation-related determinants were increased in the P301S slices. Using fluorescence microscopy, we showed target engagement of the B6 antibody to pTAU-expressing neurons and a subtle but consistent decrease in intracellular pTAU with the B6 treatment. Collectively, this tauopathy slice culture model enables measuring the extracellular and intracellular effects of different mechanistic or therapeutic manipulations on TAU pathology in adult tissue without the hindrance of the blood–brain barrier.

## 1. Introduction

Several neurodegenerative diseases, collectively termed tauopathies, are characterized by the intracellular aggregated deposits of hyperphosphorylated microtubule-associated protein (pTAU) [1]. Alzheimer’s disease (AD), frontotemporal dementia and supranuclear palsy are among these disorders, where intraneuronal pTAU starts to propagate and spreads between neurons, eventually distorting the central nervous system’s (CNS) functionality and leading to dementia [1,2,3]. These prion-like attributes, in addition to the wide array of diseases where TAU pathology is implicated, make it an appealing target for therapeutics. There are numerous clinical trials searching for cures or benefits from passive immunization, vaccination, and antibodies that target different TAU regions and phosphorylation sites [4]. In addition to their possible therapeutic effects, antibodies against pTAU could act as a diagnostic tool in magnetic resonance imaging when conjugated with magnetic nanoparticles [5,6]. These conjugates could thus have theragnostic efficacy, simultaneously facilitating diagnosis and therapy.

Mutations in the TAU gene (*MAPT*) are causative in a subset of frontotemporal dementia cases [1]. One of these, the P301S mutation, has been introduced into a transgenic mouse model (P301S mice) that expresses mutated TAU under neuron-specific elements of thy1 promoter [7]. These mice recapitulate the behavioral and pathological changes observed in familial frontotemporal dementia cases [8,9,10,11], thus enabling studying the disease’s mechanisms and treatment options.

Organotypic slice cultures are an established model system in neurodegenerative studies. Cultures are typically prepared from postnatal rodents and used widely in proteinopathy research [12]. Slice cultures preserve the anatomical hierarchy, all cell types, and cellular interactions and enable mechanistic studies without the hindrance of the blood–brain barrier. Noteworthily, slice cultures reduce the variability and number of animals needed and their suffering when compared to in vivo research. Tens of slices can be obtained from a single animal to be used as replicates, and only tissues are used instead of live transgenic animals that, in neurodegenerative studies, are often aged long, handled repeatedly and possibly develop severe phenotypes. In studying neurodegenerative diseases affecting adult and aged individuals, cultures originating from adult tissue are superior to neonatal tissue; however, comprehensive characterizations and established, repeatable viable transgenic adult tissue models are still lacking, regardless of the multiple attempts [13].

As there are, to our knowledge, no reported viable adult-originating hippocampal slice cultures for tauopathy studies, we prepared hippocampal slice cultures from adult P301S and wildtype (WT) mice and utilized this ex vivo system to study the treatment efficacy and safety of anti-pTAU antibody (B6) and magnetic nanoparticle (NP) conjugates while simultaneously characterizing the cultures. Here, we show that this model is suitable for testing pTAU-targeted treatment efficacy, initial safety, and intracellular as well as extracellular target engagement. Furthermore, with this model, we demonstrate the target engagement of the anti-pTAU treatment and the subsequent reduction in intracellular pTAU levels.

## 2. Materials and Methods

### 2.1. Animal Model and Ethics

Adult female homozygous Thy1-hTau.P301S mice [7] and wildtype controls with a CBAxC57BL6 background were obtained via collaboration with the contract research organization, reMynd (Leuven, Belgium). The animals were housed in a controlled environment (22 ± 1 °C, humidity 50–60%, and 12 h light–dark cycle) and had unlimited access to water and food. Mice were sacrificed via swift cervical dislocation in accordance with the guidelines of the European Community Council Directives 86/609/EEC. The study was approved by the Animal Experiment Board of Finland (license: ESAVI-4553-04.10.07-2016).

### 2.2. Organotypic Hippocampal Slice Cultures from Adult Mice

The slice cultures were established according to a previously described interface method [14], with some modifications and serum-free culture conditions [15,16]. First, 2- to 5.5-month-old mice were sacrificed using cervical dislocation. After decapitation, the brain was removed and immersed in an ice-cold dissection medium (DM) composed of Hibernate A (A1247501, Gibco, Waltham, MA, USA), 2% B27 supplement (17504044, Gibco), 2 mM L-glutamine (17-605E, Lonza Basel, Switzerland,), Gentamicin, and Amphotericin B (15750060, 15290018, Gibco). Hippocampi were dissected on ice, mounted onto wet filter paper (Whatman Ø55 mm, 1001-055, Cytiva, Marlborough, MA, USA), and sliced coronally to 300-µm slices with the McIlwain Tissue Chopper (The Mickle Laboratory Engineering Co., Ltd., Surrey, UK). The slices were then gently separated in fresh ice-cold DM with the help of a stereomicroscope and micro spatulas (10089-11, Fine Science Tools GmbH, Heidelberg, Germany). All slices with tears or compromised hippocampal layer architecture were discarded. In a laminar flow hood, the intact slices were mounted onto pre-wetted and warmed (37 °C) membrane inserts (PICM0RG50, Merck, St. Louis, MO, USA) in 6-well plates (140675, Nunc, Roskilde, Denmark) containing 1 mL/well of culture medium (CM, Neurobasal A (10888022, Gibco), 2% B27 supplement (17504044, Gibco), 2 mM L-glutamine (17-605E, Lonza), Gentamicin, and Amphotericin B (15750060, 15290018, Gibco)). For mounting, the slices were gently slid from the ice-cold DM onto a small spatula with a fine brush or 1000 µL pipet tip and then slid from the spatula onto an insert membrane, carefully avoiding folding and unnecessary touching. Slices from one mouse were evenly distributed throughout the plate with 5–6 slices/insert and incubated in humidified 5% CO_2_ at 37 °C. The duration of the entire protocol, from the sacrifice to the start of the incubation, was kept constant for a maximum of 30 min. The cultures were maintained with a total medium change on the day after plating, and thereafter, half of the medium was refreshed twice a week. The slices that remained unattached and without any visible growth on the outer edges (the visual estimation was conducted with a stereomicroscope) were discarded during the first seven days of culture to prevent adverse apoptotic or cytokine signaling to the other slices growing in the same well. The slices were cultured for key experiments 1 and 2 for 14 days in vitro (DIV14), but also up to DIV75 for characterization purposes.

### 2.3. Treatments

**Figure 1:**
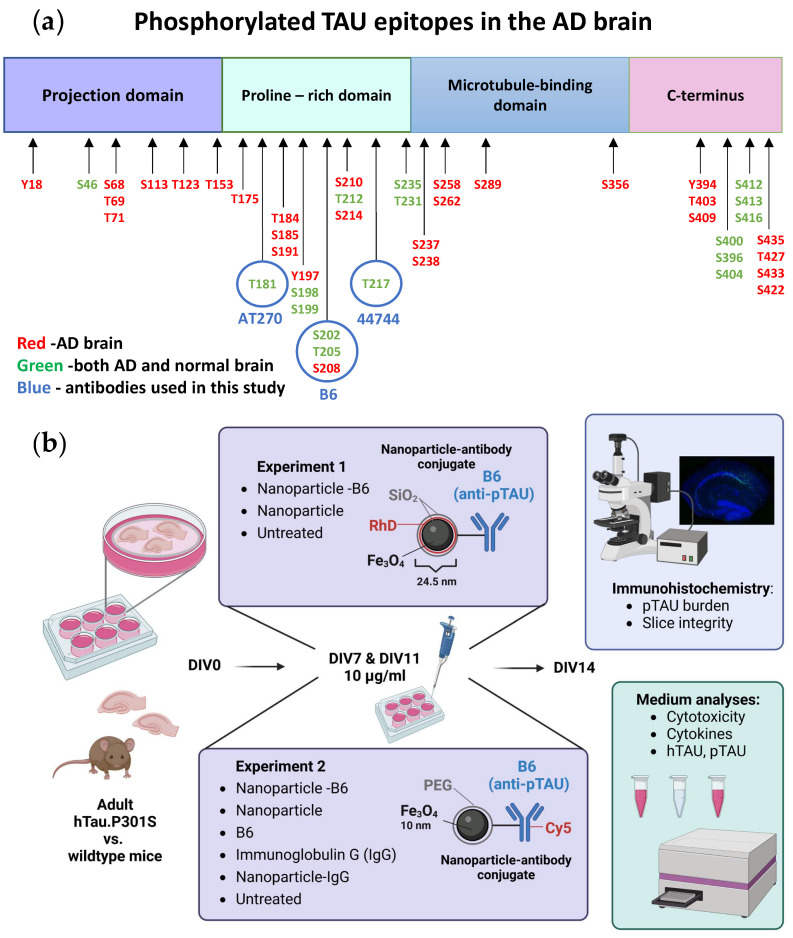
Experimental setup. (**a**) TAU phosphorylation sites [19] and antibodies used in the study (AT270 = Elisa detection antibody, B6 = studied antibody, 44,744 = antibody used in pTAU immunostainings, AD = Alzheimer’s disease). (**b**) The workflow of this study was created in BioRender.com. SiO_2_ = silicon dioxide, silica; RhD = rhodamine fluorescent compound; Fe_3_O_4_ = iron oxide; DIV = days in vitro; PEG = polyethylene glycol; Cy5 = Cyanine 5 fluorescent compound.

Custom-made monoclonal B6 antibody, specific for pTAU S202, T205, and S208 (Figure 1a), was produced in mouse hybridoma cells for the purpose of this study and has been previously used in the biochemical analyses of pTAU [17,18].

Two separate experiments with adult-originating organotypic hippocampal slice cultures were performed to evaluate the possible theragnostic effects of the different nanomaterial agents (Figure 1b). For experiment 1, the slice cultures were established from six 5-month-old WT and P301S mice. These slices were treated with 10 µg/mL silica-coated iron oxide nanoparticles (Fe_3_O_4_@SiO_2_-RhD@SiO_2_, mean size 25.4 nm) or nanoparticle-antibody conjugates (Fe_3_O_4_@SiO_2_-RhD@SiO_2_-B6) in culture medium on DIV7 and DIV12. Treatments were added on top of the slices (8 µL/slice) and into the feeding medium below the membrane inserts. The untreated (UNT) controls received the culture medium. For experiment 2, the slice cultures were established from eight 5-month-old WT and P301S mice to test the polyethylene glycol (PEG)-coated iron oxide nanoparticles (Fe_3_O_4_@PEG, core diameter 10 ± 1 nm) and nanoparticle-antibody conjugates. There were six treatment groups that consisted of groups receiving the (1) B6 antibody tagged with Cy5 (B6); (2) Cy5-tagged-B6 antibody conjugated to the nanoparticle (NP-B6); (3) nanoparticles tagged with Cy5 (NP); (4) control antibody, immunoglobulin G, tagged with Cy5 (IgG); (5) Cy5-tagged-IgG conjugated to NP (NP-IgG); and (6) UNT control group receiving a plain culture medium. The treatments were applied in the culture medium at DIV7 on top of the slices (20 µg/mL, 5 µL) as well as to the feeding medium (10 µg/mL), and at DIV11 again to the feeding medium (10 µg/mL). The experiments were ended on DIV14 by fixation of the slices. The medium samples were collected and stored during culture and at the endpoint.

### 2.4. Immunohistochemistry

The slices were collected for immunohistochemistry by rinsing the inserts and wells twice with 1 mL of PBS and fixing them with 4% PFA (2–14 h) while on the membrane, teased off the membrane with a brush and placed in 30% sucrose overnight and stored in cryoprotectant at −20 °C. Free-floating slices were stained with fluorescent secondary antibodies. Non-specific antibody binding was blocked with 3% or 5% bovine serum albumin (BSA) in Tris + Triton buffer (TBS-T, pH 7.6). To visualize the pTAU burden, the slices were incubated for 72 h with rabbit anti-pTAU T217 (1:1000, 44-744, Invitrogen, Carlbad, MA, USA) followed by goat anti-rabbit Alexa Fluor 488 (1:400, A11008, Invitrogen). To visualize the astrocytes, the slices were incubated for 48 h with rabbit anti-GFAP (1:1000, Z0334, Dako/Agilent, Santa Clara, CA, USA) followed by goat anti-rabbit Alexa Fluor 568 (1:400, A11036, Invitrogen). To visualize the microglia, the slices were incubated for 48 h with goat anti-IBA1 (1:500, ab5076, Abcam, Cambridge, UK) and rat anti-CD68 (1:5000, MCA1957, Bio-Rad, Hercules, CA, USA) followed by donkey anti-goat Alexa Fluor 568 (1:400, A11057, Invitrogen) and Donkey anti-rat Alexa Fluor 488 (1:400, A21208, Invitrogen). All primary antibody incubations were carried out in a blocking buffer at +4 °C, and the secondary antibody incubations were in plain TBS-T at room temperature for 3–5 h. Nuclei were stained with DAPI (1:1000, 15 min) in all slices. The slices were mounted on gelatin-coated slides with the Vectashield Hard Set (H-1400, Vector laboratories, Burlingame, CA, USA). The control sections without primary antibodies were processed simultaneously to monitor for unspecific signals.

### 2.5. Microscopy and Image Analysis

The light microscopy images during the culture (live images DIV0-75) were obtained with a Leica MZ6 microscope (0.5× objective) equipped with the MC120 camera (Leica Microsystems GmbH, Wetzlar, Germany). Fluorescence wide-field images were obtained with a Leica DM6B-Z microscope with a K5-14400955 camera and Thunder image processing (Leica Microsystems GmbH). Confocal fluorescence images were obtained with a Zeiss Axio Observer microscope equipped with the LSM700 or LSM800 confocal module (Carl Zeiss Microimaging GmbH, Jena, Germany). The light, laser, and detector settings were kept constant for all samples for each specific immunostaining. Quantitative image analysis was carried out using the Fiji ( ImageJ 1.53c) software [20]. The Z-stacks from each channel were maximum-intensity projected, background subtracted (using the rolling ball algorithm), and Gaussian blurred. To quantify the phosphorylated TAU for experiment 1, the pTAU and DAPI channels were thresholded with the Moments threshold (pTAU 5907/65535, DAPI 11447/65535), and the regions of interest (ROI) selected were with the Analyze Particles function. For experiment 2, the pTAU channel was thresholded with the Triangle automatic threshold, the DAPI channel with the Li automatic threshold, and segmented with Watershed prior to the Analyze Particles function.

### 2.6. Medium Analyses

The conditioned medium was collected on fixed time points when the medium was refreshed during the culture period and at the end of the study. The samples were stored as frozen aliquots, and most analyses were run with sample thawed only once. To facilitate the target engagement study of extracellular pTAU to B6 antibody, an approximate ¾ volume of the medium samples collected after the treatment application (DIV11, DIV14) were subjected to 2 h of magnetic separation with a neodymium 200 kg strength magnet (Supermagnete, Gottmadingen, Germany). This magnetic fractionation resulted in three separate medium sample types: (1) total medium sample; (2) supernatant (non-magnetic fraction, collected after 2 h on and while on the magnet); and (3) magnetic fraction (Mg, washed twice with HEPES 10 mM + Glucose 0.01% on top of the magnet and diluted in HEPES 10 mM + Glucose 0.01% after removing the magnet). ELISA kits (Innotest Phospho-TAU (181P, AT270), 81574, Innotest hTAU Ag, 81572, Fujirebio, Gent, Belgium) were used to measure the pTAU and total TAU levels from the total and magnetic fraction of the conditioned medium. The protein concentrations for normalizations were measured from the total fraction of the conditioned medium with the Pierce BCA Protein Assay Kit (23227, Thermo Scientific, Waltham, MA, USA). The Interleukin 6 (IL-6), tumor necrosis factor α (TNFα), neurofilament light (NFL) and lactate dehydrogenase (LDH) levels were measured from the supernatant fraction of the conditioned medium with the IL-6 Mouse ELISA Kit (88-7064-22, Invitrogen), TNFα Mouse ELISA Kit (88-7324-22, Invitrogen), Mouse Neurofilament Light Polypeptide ELISA Kit (MBS2021074, MyBioSource, San Diego, CA, USA) and Cytotoxicity Detection Kit (11644793001, Roche Diagnostics, Risch-Rotkreuz, Switzerland), respectively. All analysis kits were used according to the manufacturer’s recommendations. Soluble TREM2 (sTREM2) was measured from the supernatant fraction of the conditioned medium using a previously described in-house ELISA assay (Zhong et al., 2019), with minor modifications described as follows: High bind microplates (9018, Corning Incorporated, Corning, NY, USA) were used, and the samples were diluted at a 1:5 ratio in assay buffer (1% BSA and 0.05% Tween 20 in PBS) for the measurement. Recombinant mouse TREM2 (50149-M08H, Sino Biological Europe GmbH, Eschborn, Germany) was used as a standard, and the detection antibody was incubated for 1 h at room temperature. After 15 min of development with the substrate, the reaction was stopped with the 1 M H_3_PO_4_ solution, and the plate was read at 450 nm with a Tecan Infinite M200 Microplate Reader (Tecan, Männedorf, Switzerland).

### 2.7. Statistical Analyses

IBM Statistics, version 27, was used for the statistical analyses, and GraphPad Prism 9 was used for the visualization of the results. The results were analyzed using the one-way or two-way ANOVA or ANOVA for repeated measures with the genotype (WT vs. P301S) and treatment as the between-subject factors. If there was a significant difference in ANOVA, the Bonferroni post hoc test was run to reveal the differences between specific groups. The results are shown as the mean ± standard deviation (SD) and as individual data points that correspond individual slices (immunohistochemical data) or wells (culture medium data). *p*-values < 0.05 are considered statistically significant.

## 3. Results

### 3.1. Adult Tissue-Originating Organotypic Hippocampal Slice Cultures Retain All Cell Types and Intact Structural Hierarchy during Culturing

**Figure 2:**
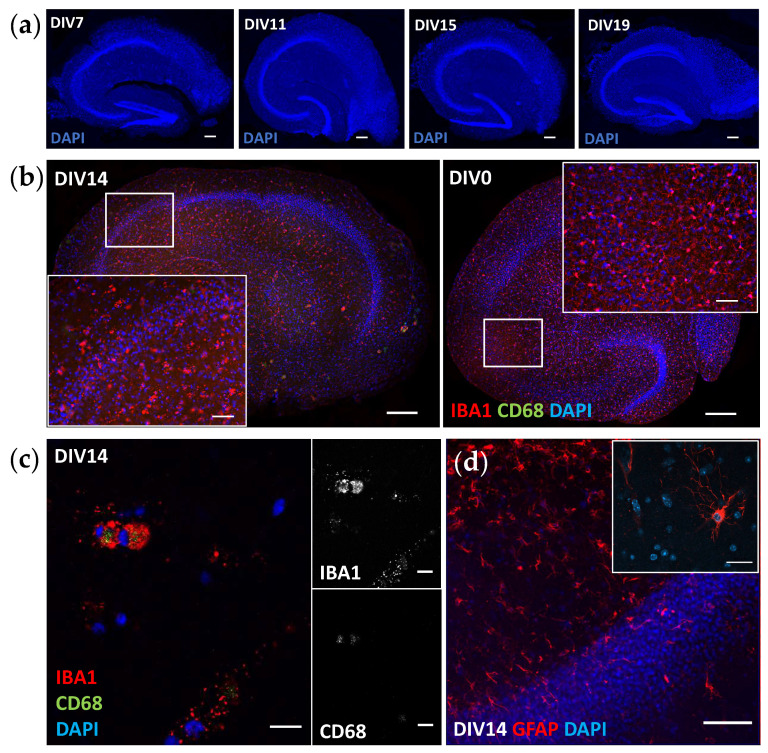
Structural and cellular characterization of adult hippocampal slice cultures. (**a**) The granular cell layer remained intact throughout the culture (scale bar 20 µm). Nuclei are shown in blue (DAPI). (**b**) Microglia are evenly distributed in the cultured slice and have a droplet-like appearance when visualized with microglia marker IBA1 (red) at DIV14 (left panel). For comparison, the DIV0 slice, fixed without plating, is shown (right panel). Scale bars 200 µm (whole slice) and 50 µm in close-up. (**c**) High-resolution image of functional microglia positive for IBA1 (red in merged image) and macrophage lysosomal marker CD68 (green in merged image) at DIV14. Scale bars 10 µm. (**d**) GFAP-positive astrocytes (red) at DIV14, scale bars 50 and 20 µm. DIV = days in vitro. All shown images are from wildtype tissue.

**Figure 3:**
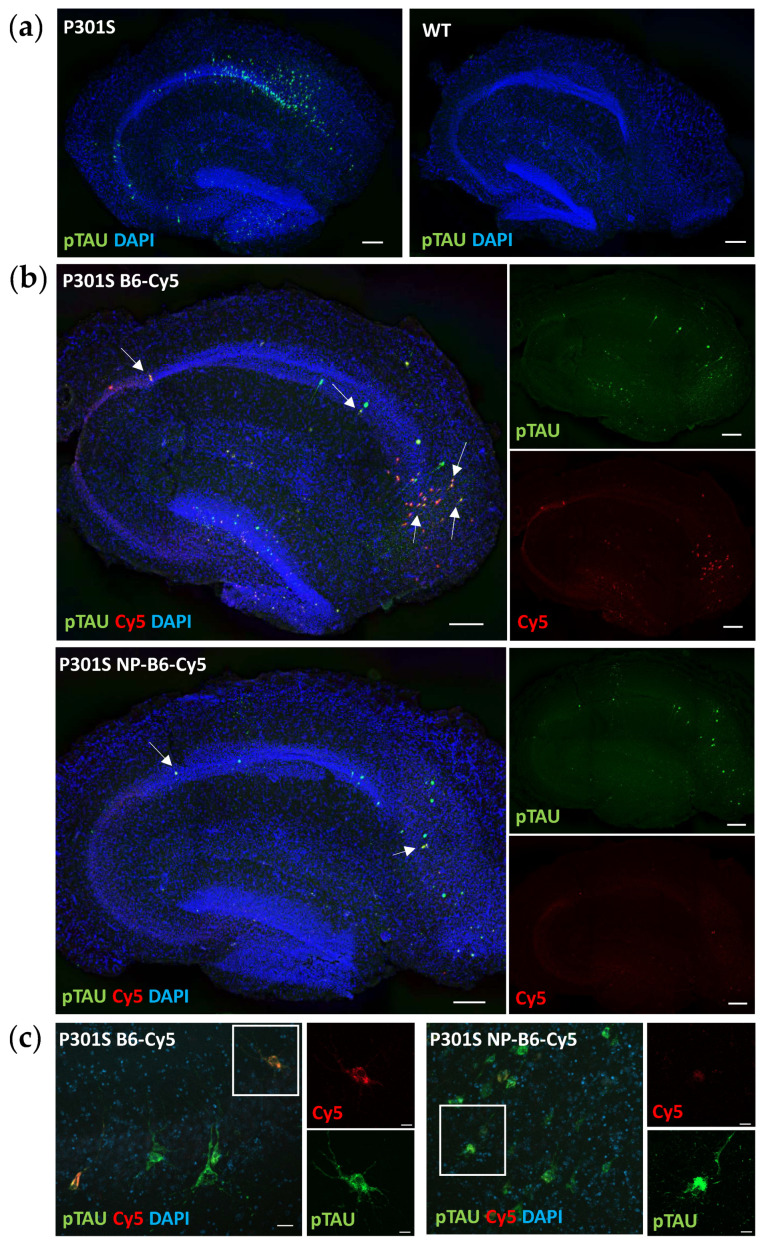
P301S slices have pTAU-positive neurons, and the B6 antibody bound these when added during culture. (**a**) In P301S slices, pTAU+ neurons (green) reside throughout the granular cell layer, while in wildtype (WT) slices, there are none (DIV14, scale bars 200 µm). (**b**) Cy5-tagged-B6 antibody (B6-Cy5, red) and PEG-coated nanoparticle (NP) conjugated with B6 (NP-B6-Cy5, red) co-localize (white arrows) with pTAU+ neurons (green) (scale bars 200 µm). NP conjugated to B6 decreases target engagement. (**c**) High-resolution image of B6 target engagement to pTAU-positive neurons, scale bars 20 µm and 10 µm. Nuclei (DAPI) are shown in blue in all samples.

Approximately 65–70% of the slices were attached to the membrane and began growing from the edges during the first seven days of culture. This success rate did not differ between the genotypes (experiment 1: P301S 64% and WT 58%; experiment 2: P301S 73% and WT 73%). The serum-free protocol used retained an intact structural hierarchy of the hippocampus, and the granular cell layer was clearly visible and anatomically comparable to in vivo when the nuclei were stained with DAPI (Figure 2a). The layered structure of the hippocampus remained intact and visible without any specific staining up to DIV75 and also when it was imaged during the culture with a stereomicroscope (Figure S1). At DIV14, the microglia that were positive for IBA1 and CD68 were present evenly throughout the slices (Figure 2b,c), and an antibody against GFAP clearly visualized astrocytes (Figure 2d). The staining pattern of IBA1 changed from a ramified appearance on DIV0 to droplet-like on DIV14 (Figure 2b). Importantly, inside IBA1-positive microglia, there were clear CD68-positive lysosomes, indicating the metabolic functionality of microglia in the cultured slices (Figure 2c). Neuronal survival was visualized with a pTAU staining pattern on the P301S slices, where a subset of neurons in the granular cell layer was strongly positive for hyperphosphorylated TAU (Figure 3a). In addition, granular cell layer neurons were visualized with B6 (anti-pTAU) antibody tagged with the fluorochrome Cy5 applied on the slices during culturing (Figure 3b). Furthermore, neuronal survival was evident from the measurable levels of pTAU in the P301S-slice conditioned medium(described in detail in the results Section 3.5), as the expression of mutated TAU in P301S mice is neuron-specific [7,9]. Taken together, these results validate that this adult-originating organotypic slice culture method is suitable for studying all central nervous system resident cell types and their interactions during at least two weeks and likely up to several months.

### 3.2. P301S Slices Have pTAU-Positive Neurons, and B6 Antibody Binds to pTAU Structures during Culturing

**Figure 4:**
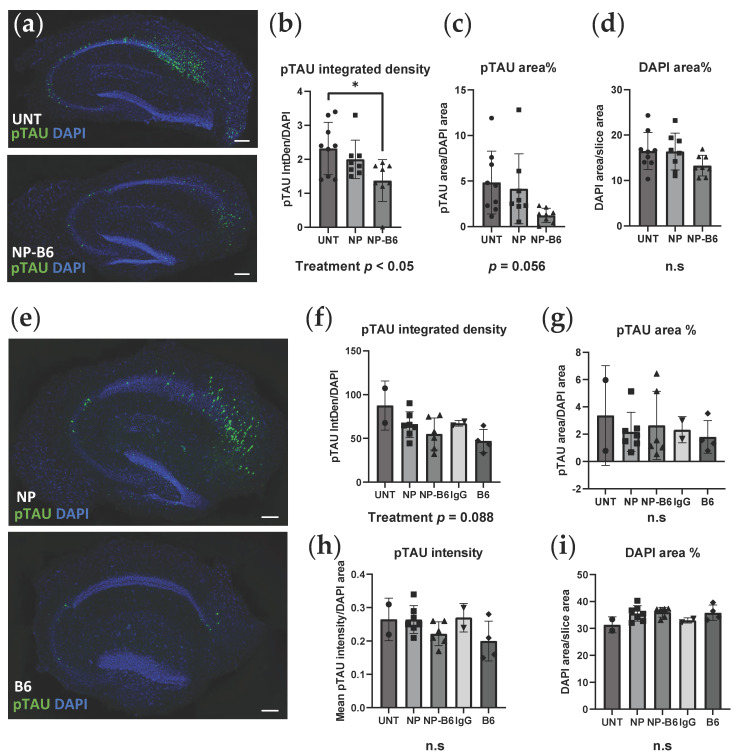
B6 antibody, administered during culture, decreased the pTAU signal in both experiments (significantly in experiment 1 with silica-coated nanoparticle (NP)-conjugate). (**a**) Representative images of P301S slices treated with plain conditioned medium (UNT) and NP-B6 antibody conjugate stained with pTAU antibody (green) and DAPI for nuclei (blue). (**b**) Quantification of pTAU signal in P301S slices of experiment 1. (**c**) Quantification of pTAU-positive area. (**d**) Quantification of DAPI area as a proxy for viability in P301S slices. (**e**) Representative images of P301S slices treated with PEG-coated NPs and B6 antibody stained with pTAU antibody (green) and DAPI for nuclei (blue). (**f**) Quantification of pTAU signal in P301S slices in experiment 2. (**g**) Quantification of pTAU-positive area. (**h**) Quantification of pTAU signal intensity. (**i**) Quantification of DAPI area. Scale bars 200 µm. Mean ± SD, individual data points correspond to individual slices, for (**b**–**d**) *n* = 8–9, ANOVA, * *p* < 0.05, Bonferroni, for (**f**–**i**) *n* = 2–7, ANOVA. Key: UNT = untreated; NP = nanoparticle; NP-B6 = nanoparticle-B6 antibody conjugate; IgG = immunoglobulin G; B6 = anti-pTAU antibody.

The immunofluorescence staining for hyperphosphorylated TAU (T217) revealed that there were pTAU-positive neurons throughout the granular cell layer in the P301S slices, most prominently in the cornu ammonis 1 (CA1) area, while the WT slice neurons had no pTAU signal (Figure 3a). The B6 antibody targeted the pTAU-positive neurons when applied during the culture period (Figure 3b,c), as shown by co-localization of the pTAU signal from antibody staining with the Cy5 signal of tagged B6. PEG-coated nanoparticle conjugate seemed to hinder the B6 target engagement, as there were fewer neurons visible on the Cy5 channel in the conjugate-treated slices (visual estimation) when compared to the B6-treated slices, even though there were no differences in pTAU positivity between these groups (Figure 4e–h). Silica-coated nanoparticle-B6 antibody conjugate (experiment 1) did not co-localize with the pTAU (T217)-positive neurons (Figure S2).

### 3.3. B6 Anti-pTAU-Treatment Reduces Intracellular pTAU Levels in P301S Slices

To assess the treatment effects on intracellular pTAU levels, the P301S slices were immunofluorescence stained for pTAU (T217). The image analysis revealed that the integrated density (a real-world correlate of the amount of probe) of pTAU decreased in the silica-coated nanoparticle-B6 conjugate-treated slices compared to the controls (Figure 4b, treatment *p* < 0.05, experiment 1). Additionally, the pTAU-positive area decreased with borderline significance in conjugate-treated slices (Figure 4c, treatment, *p* = 0.056). The DAPI area was equivalent in all groups, indicating similar viability (Figure 4d). Consistent with the results from experiment 1, the B6 antibody and PEG-coated nanoparticle-B6-conjugate showed a decreasing trend in the integrated density of pTAU in the treated slices (Figure 4f, *p* = 0.088, experiment 2). Additionally, the pTAU area and mean intensity (the concentration of the probe) of the pTAU signal were the lowest in the B6-treated groups (Figure 4g,h, nonsignificant). The DAPI area was equivalent in all groups, indicating similar viability (Figure 4i) in experiment 2 as well. Taken together, these results indicate a subtle but consistent reduction in intracellular pTAU with B6 treatment in the cultured hippocampal P301S slices.

### 3.4. Cytotoxicity and Cytokine Release Is Increased in P301S Slices as Compared to Wildtype Slices

**Figure 5:**
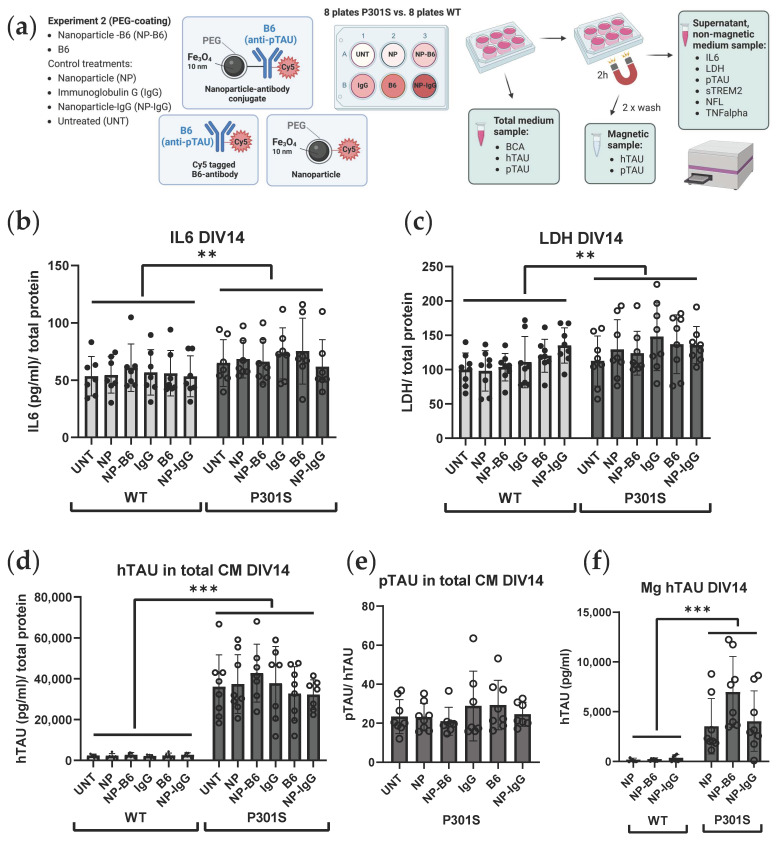
Examination of the P301S slice medium revealed increased inflammation, cytotoxicity, and TAU phosphorylation compared to the wildtype (WT) slice medium. (**a**) Experimental setup depicting treatment groups, conjugate compositions, and magnetic fractionation of the culture medium (CM) for following analyses. Fe_3_O_4_ = iron oxide; PEG = polyethylene glycol; Cy5 = Cyanine 5 fluorescent compound. Created in BioRender.com. (**b**) Interleukin 6 (IL6) levels in relation to total protein content at DIV14. (**c**) Lactate dehydrogenase (LDH) levels in relation to total protein at DIV14. (**d**) Extracellular levels of hTAU at DIV14 in relation to total protein. (**e**) Levels of phosphorylated TAU (pTAU) at DIV14 for P301S slices. The levels in the WT slice medium were undetectable and thus not shown. (**f**) hTAU levels in the magnetically separated medium fraction (Mg) of the NP-treated samples. Mean ± SD, individual data points correspond to samples from individual wells, *n* = 7–8. ** *p* < 0.01, *** *p* < 0.001, two-way ANOVA. DIV = days in vitro.

To characterize the inflammatory processes in the slice cultures, the levels of cytokines IL6, TNFα, and sTREM2 were measured from the conditioned medium. The IL6 levels in the conditioned medium decreased from DIV11 to DIV14 (Figure S3a, DIV *p* < 0.001) and were higher in the P301S medium than in the WT medium (Figure S3a, genotype *p* < 0.05) when both time points were analyzed combined in experiment 2. Additionally, when explored separately only at DIV14, the P301S genotype increased the IL6 levels compared to the controls (Figure 5b, genotype *p* < 0.01). The TNFα levels were below the detection limit, regardless of the genotype. The results of experiment 1, regarding the levels of the cytokines, were consistent: The P301S genotype also increased the IL6 levels at DIV14 in that sample set (Figure S4a, *p* < 0.05). The sTREM2 levels were on a borderline threshold of the detection limit by the in-house ELISA used, thus preventing the analysis of the results. Yet, all the WT samples were comparable to blank sample having signal below the detection limit of the plate reader used, whereas almost all of the P301S medium samples had some signal detected. Thus, a more sensitive analysis method might reveal differences in the sTREM2 levels and microglial activity status in the P301S slices compared to the controls.

The LDH levels were measured from the conditioned medium as a proxy for cytotoxicity. The LDH levels decreased from DIV11 to DIV14 in the WT slice medium, whereas they significantly increased in the P301S slice medium (Figure S3b, DIV × genotype *p* < 0.01) in experiment 2. NP-IgG increased the LDH levels compared to the UNT slices when both time points were analyzed combined (Figure S3b, treatment *p* < 0.05, UNT vs. NP-IgG *p* < 0.05). The LDH release into the medium at DIV14 was significantly increased in the P301S slices compared to the WT slices (Figure 5c, *p* < 0.01) when the DIV14 results were examined separately from DIV11. Again, here, the results of experiment 1 were consistent with the aforementioned experiment 2 results and supported the finding that the P301S genotype leads to increased cytotoxicity on DIV14 (Figure S4b, genotype *p* < 0.05).

Neurofilament light was measured from the conditioned medium to evaluate the axonal damage. Neurofilament light levels in the conditioned medium were below the detection limit of the ELISA kit used, regardless of the genotype, indicating that there was no gross axonal damage in the slices at the end of the culture period.

### 3.5. TAU Secretion and Phosphorylation Is Increased in P301S Slices as Compared to Wildtype Slices

**Figure 6:**
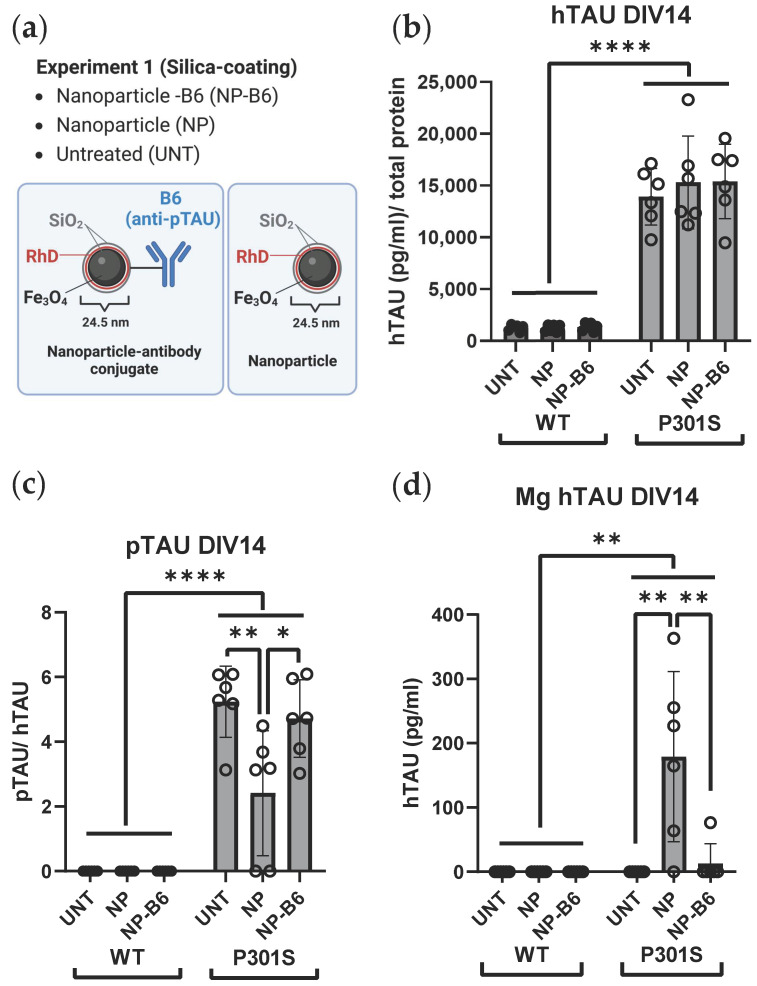
TAU secretion and phosphorylation were consistently increased in the P301S slice medium compared to the wildtype (WT) slice medium, and the extracellular target engagement to pTAU remained inconclusive for both nanomaterial-B6 conjugates tested in the slice culture setting. (**a**) Experimental setup depicting treatment groups and nanoparticle (NP) and NP-B6 conjugate composition in experiment 1. SiO_2_ = silicon dioxide, silica; RhD = rhodamine fluorescent compound; Fe_3_O_4_ = iron oxide. Created in BioRender.com. (**b**) The hTAU levels in relation to total protein content at DIV14. (**c**) pTAU levels in relation to total TAU at DIV14. Bare silica-coated NPs appeared to reduce pTAU levels. (**d**) hTAU levels at DIV14 in magnetically pulled-down medium fraction (Mg). Bare NPs seemed to trap some pTAU. Mean ± SD, individual data points correspond to samples from individual wells, *n* = 6, * *p* < 0.05, ** *p* < 0.01, **** *p* < 0.0001, two-way ANOVA, Bonferroni. DIV = days in vitro.

To assess the amount of extracellular TAU and pTAU in the cultures, the hTAU and pTAU levels were measured from the conditioned medium. The P301S genotype significantly increased hTAU secretion (Figure 5d, genotype *p* < 0.001) and TAU phosphorylation (Figure 5e, genotype *p* < 0.001) in experiment 2. The pTAU levels in the conditioned medium from WT slices were below the detection limit and thus are not shown. Experiment 1’s results are consistent with the aforementioned: the P301S genotype increased hTAU secretion (Figure 6b, genotype *p* < 0.0001) and TAU phosphorylation (Figure 6c, genotype *p* < 0.0001), which is also in that sample set.

### 3.6. Nanomaterial Conjugate or B6 Do Not Elicit Harmful or Beneficial Changes Measurable in Medium

The PEG-coated NPs (experiment 2) did not elicit any inflammatory or cytotoxic responses in the ex vivo organotypic culture (Figure 5b,c). Only the NP-IgG treatment increased the LDH release compared to the untreated slices when the results from both DIV11 and DIV14 were combined (Figure S3b, *p* < 0.05). This effect, however, was not significant when the DIV14 results were examined separately from DIV11 (Figure 5c). The PEG-coated NPs did not influence TAU secretion or phosphorylation (Figure 5d,e) either. Similarly, bare antibodies (B6 and IgG) had no effect on the extracellular targets measured from the conditioned medium. Magnetic separation (Figure 5a) of the conditioned medium did not show a clear target engagement of B6 to extracellular TAU (Figure 5f). The levels of pTAU from the magnetic fraction were undetectable with the ELISA kit used. However, the NP-B6 conjugate-treated P301S slice culture samples showed a trend towards increased levels of hTAU as compared to the slice cultures receiving other NP treatments (Figure 5f, *p* = 0.076), implicating that some pTAU might have been caught with B6 and pulled into the magnetic fraction by conjugated iron-cored NP using magnetic separation. Another indirect way to measure NP-B6-bound pTAU in the magnetic fraction is to calculate the difference in pTAU concentrations between the total medium and supernatant. This, however, was not different between the treatment groups and thus did not show a measurable decrease in NP-B6-bound pTAU after the magnetic separation (Figure S5). As expected, the hTAU levels in the magnetic fraction were significantly higher in the P301S samples when compared to the wildtype samples.

In line with the above data, the silica-coated NP-B6 conjugate (experiment 1) did not elicit an increase in cytotoxicity or IL6 cytokine levels (Figure S4) or a reduction in TAU secretion or phosphorylation at DIV14 (Figure 6b,c). However, surprisingly, the bare silica-coated NPs decreased extracellular pTAU levels in the conditioned medium from experiment 1 when compared to the untreated (Figure 6c, *p* = 0.007) and NP-B6-treated slices (Figure 6c, *p* = 0.031). This is in line with the hTAU levels in the magnetic fraction as well; magnetically pulled-down conditioned medium fractions from NP-treated slices had more hTAU than the untreated (Figure 6d, *p* = 0.001) and NP-B6 treated (Figure 6d, *p* = 0.003). These results together indicate that silica-coated NPs might have bound or trapped hyperphosphorylated TAU species, probably in an unspecific manner. This is unexpected, as the same NP conjugated to B6 did not appear to bind extracellular pTAU. One explanation could be the behavior of this silica-coated NP-B6 conjugate, as it was extremely prone to aggregate in the biological liquids tested and thus might not have been able to bind pTAU in the aggregated form. This aggregation tendency and missing target engagement, according to the pTAU immunohistochemistry, is the reason why we here focus mainly on the PEG-coated conjugates (experiment 2). It is hard to imagine that the decreasing effect the bare particles had on extracellular pTAU levels would be translatable to in vivo and warrants more studies.

Taken together, the B6 antibody and B6-nanomaterial conjugate treatments appear non-harmful but inefficient regarding the extracellular TAU release and phosphorylation in the slice cultures. Moreover, the target engagement of B6 to extracellular TAU remains inconclusive.

## 4. Discussion

The purpose of this study was to set up and characterize an ex vivo tauopathy slice culture model and test the theragnostic applicability of magnetic nanoparticles conjugated to antibody against pathogenic hyperphosphorylated TAU in this model. Slice cultures can function as a bridge between the in vitro and in vivo models, whose benefits include retaining multicellular crosstalk and excluding the limitations arising from the blood–brain barrier. Adult over neonatal tissue was chosen as a starting material for the slice cultures, as it is more representative of human tauopathies that typically plague adult and aged individuals. Adult-originating slices are also more suitable for studying the tissue penetrance of therapeutics, as they do not thin [21] to quasi-monolayers as the postnatal slices do [14,22].

The culturing of adult slices past an acute phase was long deemed impossible, regardless of extensive culture condition optimization [21,23], until the withdrawal of serum from the culture medium was found to be essential to adult slice viability [15,16,24]. With this information at hand, we succeeded in establishing viable adult-originating hippocampal slice cultures from mice carrying the P301S *MAPT* mutation, causative for frontotemporal dementia, for long-term tauopathy studies. According to our data, these cultures preserve all brain-resident cell types and manifest intraneuronal pTAU accumulation as well as extracellular pTAU release. This extracellular and easily accessible pool is particularly convenient for studying the effects of therapeutic manipulations on TAU spread and propagation.

The distribution of the intraneuronal accumulated pTAU detected in cultured hippocampal P301S slices was comparable to what has been previously reported in vivo around the age of 5 months [9,25]. Neurons positive for pTAU (T217) resided throughout the hippocampal granular cell layer after two weeks in culture and were visually estimated to be the most abundantly present in the CA1 area, which is well in line with the AT180 (T231) immunoreactivity shown at the ages of 4 and 6 months in mice. [25]. Intraneuronal pTAU accumulation continues in slice culture settings and appears to be somewhat accelerated when compared to in vivo as there were only a few neurons positive for AT8 (S202, T205) or AT100 (T212, S214) in the P301S mouse hippocampus at 5.5 months (manuscript in preparation), which was the age of the P301S mice used to generate the slices in this study. Neuronal targets beyond pTAU were not in the scope of this study but should be examined in detail in the future. This slice culture model should facilitate studying changes in synaptic components [12] and dendritic spines [26]. Additionally, electrophysiological studies can reveal changes in neurotransmission [26,27].

Microglial contribution to neurodegeneration is regarded as central, yet it is not known whether microglial hypo- or hyperactivation, or senescence wreaks havoc in the brain. Therefore, models that enable mechanistic studies on aged and genetically challenged microglia, together with other CNS cells, are pivotal in furthering our understanding of the underpinnings of neuroinflammation. There are only a few reports on microglia in adult-originating slice cultures [13], and some state that microglia are not evident after DIV1 when the slices are cultured in a serum-containing medium [21]. Only two studies so far have reported the presence of microglia in adult slices [28,29], but the images shown leave some doubt of target specificity (IBA1) to the actual microglia and make comparisons to our immunohistological data difficult. In addition, the culture conditions and slice generation used in the previous publications differed from our protocol. Nevertheless, we have now shown that microglia are present in adult-originating slices and, more importantly, are functional after two weeks in culture. The microglia in our slices have clear CD68-positive lysosomes, indicating phagocytic capacity and active metabolism. The unexpected droplet-like appearance of IBA1 is most likely due to culturing conditions optimized for the more sensitive neurons [16]. Microglia are highly reactive, and thus, it is understandable if the microglia show deviant staining patterns when cultured in different media [30] and experience challenges related to culture preparation and maintenance, such as mechanical slicing and culturing on air–liquid interfaces. According to our data, IBA1 is not the optimal staining target in this system if the focus is on microglial morphology. Further studies will show the best microglial markers and exactly how useful this model is for studying inflammatory processes, yet there is a high promise. It has been previously shown that pTAU aggregation induces disease-associated microglia phenotypes [9] and increases astrogliosis [10] in P301S mice. Based on this, one might expect that there is also a probable inflammatory phenotype present in the P301S slices, and this needs to be studied further. Single-cell RNA sequencing to reveal the molecular signatures of the microglia in the slices [31] and identify the differences in immunohistochemical staining for CD68 and GFAP between the genotypes are the obvious next steps.

In P301S mice, memory deficits coincide with the appearance of S202 pTAU and a reduction in dendritic spine density [25], which make this transgenic model an especially suitable source of tissue to establish slice cultures and to study the effects of an antibody against the TAU phosphorylation sites S202, T205, and S208 (Figure 1a). Indeed, the B6 antibody, when added twice during the culture, could reduce the neuronal pTAU levels in P301S slices. Moreover, target engagement and tissue penetrance of B6 and PEG-coated NP-B6 conjugate to pTAU-positive neurons during the culture period inside the tissue were proven. The mechanism of neuronal pTAU reduction via B6 antibody was not studied here, but since B6 was shown to bind to the neurons in experiment 2, it is likely that TAU aggregate disassembly and degradation within the lysosomal system was promoted [32]. It is also possible that B6 initiated a cellular danger response by recruiting the cytosolic Fc receptor tripartite motif protein 21 (TRIM21) to enhance the proteasomal degradation of pTAU [33,34]. When internalized, B6 could also act to sequester the pTAU assemblies and prevent their release from the neuron [4]. However, this does not seem likely, as there was no significant reduction in the medium pTAU levels with the B6 treatment. As mentioned in the results section, the silica-coated NP-B6 conjugate was extremely prone to aggregate, which might be the reason why there was no visible target engagement with this conjugate. The aggregation possibly resulted in the formation of such a large complex that was unable to penetrate the tissue and masked some of the antibody binding sites. This conjugate, however, did reduce the pTAU signal in immunohistochemistry without proven target engagement. The mechanism underlying this reduction remains elusive; however, the finding suggests that co-localization of the antibody with pTAU-positive neurons is not mandatory for reaching a therapeutic effect, at least in this ex vivo setting. Because the silica-coated NP-B6 conjugate was unable to enter the neurons, the effect was likely mediated by an extracellular mechanism. The extracellular effects might arise from the antibody-mediated sequestering of TAU aggregates, assembly interference, and promotion of microglial phagocytosis, leading to reduced TAU spread between neurons [4]. It was recently shown that in P301S mice, microglia respond to neuronal pTAU aggregation and internalize pTAU, which was shown to co-localize with swollen lysosomes [9]. Microglial activation and their exact role in the pTAU reduction observed with the B6 treatment in the P301S slices warrant more studies. However, the finding that pTAU was reduced in neurons without a clear target engagement, is interesting and suggests that B6 can act in both extracellular and intracellular compartments and through more than one pathway, implicating that B6 could have clinical promise, as this multifaceted action might be needed for clinical efficacy [35].

The nanoparticles or particle conjugates had no adverse effects in the adult slice culture model. Only the PEG-coated NP conjugated to IgG increased the LDH levels when the DIV11 and DIV14 results were analyzed together with repeated measures ANOVA. However, this effect was not significant when only the DIV14 data were examined. Moreover, bare particles or particles conjugated to B6 or IgG alone did not induce cytotoxicity.

B6 target engagement to extracellular pTAU could not be shown in this study. This might be due to the limited sensitivity of the ELISA measurement. Alternatively, the freezing and thawing of the medium samples after sample collection might have broken the interaction between the B6 and pTAU monomers/oligomers, thus disabling the magnetic pull-down of B6-bound pTAU. The ELISA kit available should have been suitable (pTAU T181) to detect B6 (pTAU: S202, T205, S208)-bound pTAU, as the antibody target site should not have been masked with B6. The vast majority of TAU and pTAU are intracellular [4]; however, studying the extracellular pool of pTAU, which is capable of spreading between cells, is also important. In a slice culture model, this pool is easily accessible. For better utilization of this model for extracellular target engagement studies, the abovementioned hurdles need to be overcome. Single-molecule array (Simoa) [36] measurements would increase the sensitivity. Furthermore, the medium samples should be magnetically fractionated, in the case of the magnetic cored conjugates, prior to freezing and being aliquoted to avoid repeated freeze–thaw cycles, and perhaps (preferably), analyzed fresh without freezing the samples.

This multicellular ex vivo model system enables the analysis of a vast array of experimental targets. Medium-based analyses allow examination of the extracellular effects of the targets. In the case of research related to tauopathies, this is especially important, as it enables the evaluation of the effects of TAU spreading. Tissue-based analyses help to dissect the intracellular effects from extracellular ones, potentially revealing mechanisms of TAU toxicity and seeding. In slice cultures, the tissue is easily accessible for live imaging and electrophysiological recordings, and, as mentioned previously, the blood–brain barrier does not hinder the access of the studied agents to the tissue. No model system is perfect, and there are also limitations with this experimental setup. Slice cultures are void of vascular circulation, although preserved vascular cells enable studies on limited aspects of vascular function [13,28]. Slicing of the tissue induces axotomy; therefore, many important connections are lost, and blood–brain barrier-related studies are not possible. Despite these limitations, the adult tissue-originating slice culture model from the P301S tauopathy model mice is a promising system for screening therapeutic agents, unraveling their mechanisms of action, and dissecting their cell-specific effects.

## Data Availability

Not applicable.

## Supplementary Materials

The following supporting information can be downloaded at: https://www.mdpi.com/article/10.3390/cells12101422/s1, Figure S1: Adult-originating mouse hippocampal slice cultured for 75 days, Figure S2: Silica-coated nanoparticle conjugated with B6 antibody does not co-localize with pTAU positive neurons. Figure S3: P301S slice medium IL6, and LDH levels change during culture in different patterns compared to wildtypes. Figure S4: IL6 and LDH increase in the P301S medium is consistent in both experiments. Figure S5: Calculated pTAU difference with magnetic pulling in experiment 2.
