# Supplementary material for: Organotypic Hippocampal Slice Cultures from Adult Tauopathy Mice and Theragnostic Evaluation of Nanomaterial Phospho-TAU Antibody-Conjugates
Source: Cells. 2023 May 18;12(10):1422. doi: 10.3390/cells12101422 (PMC10216823; doi:10.3390/cells12101422)
Supplement: Supplementary file 1 [file cells-12-01422-s001.zip › cells-2386107-supplementary.pdf]

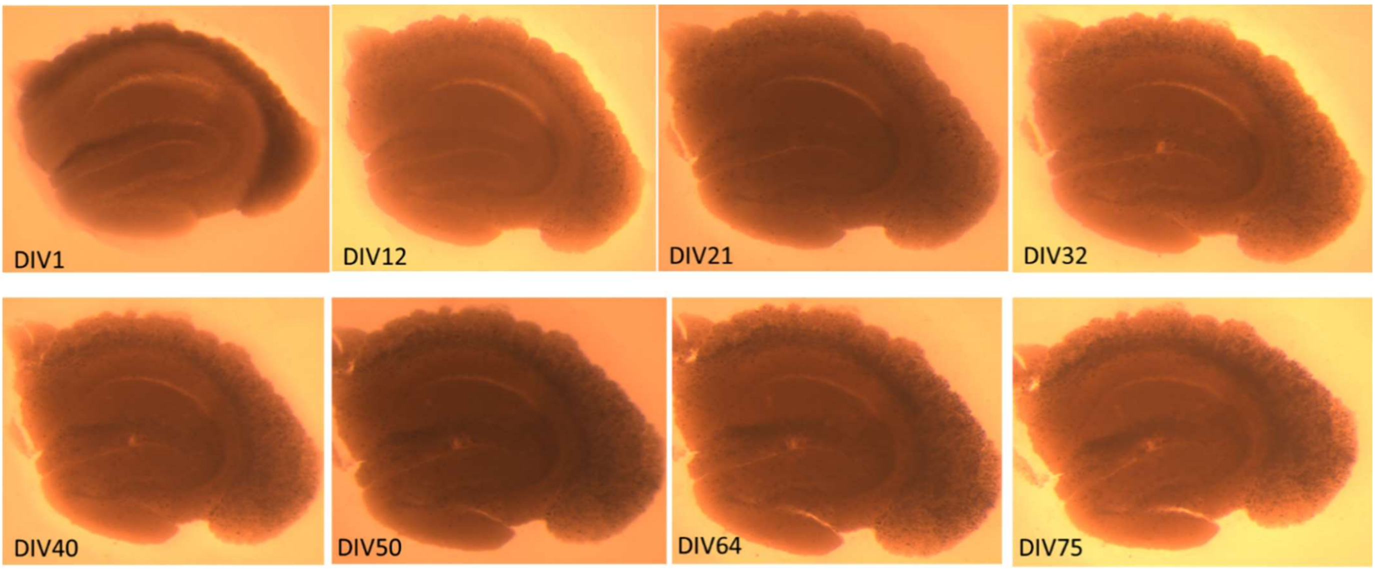

Supplementary Figure S1. Adult-originating mouse hippocampal slice cultured for 75 days. Hippocampal layers stay visible throughout culture. Imaged live with stereomicroscope. DIV = days *in vitro*.

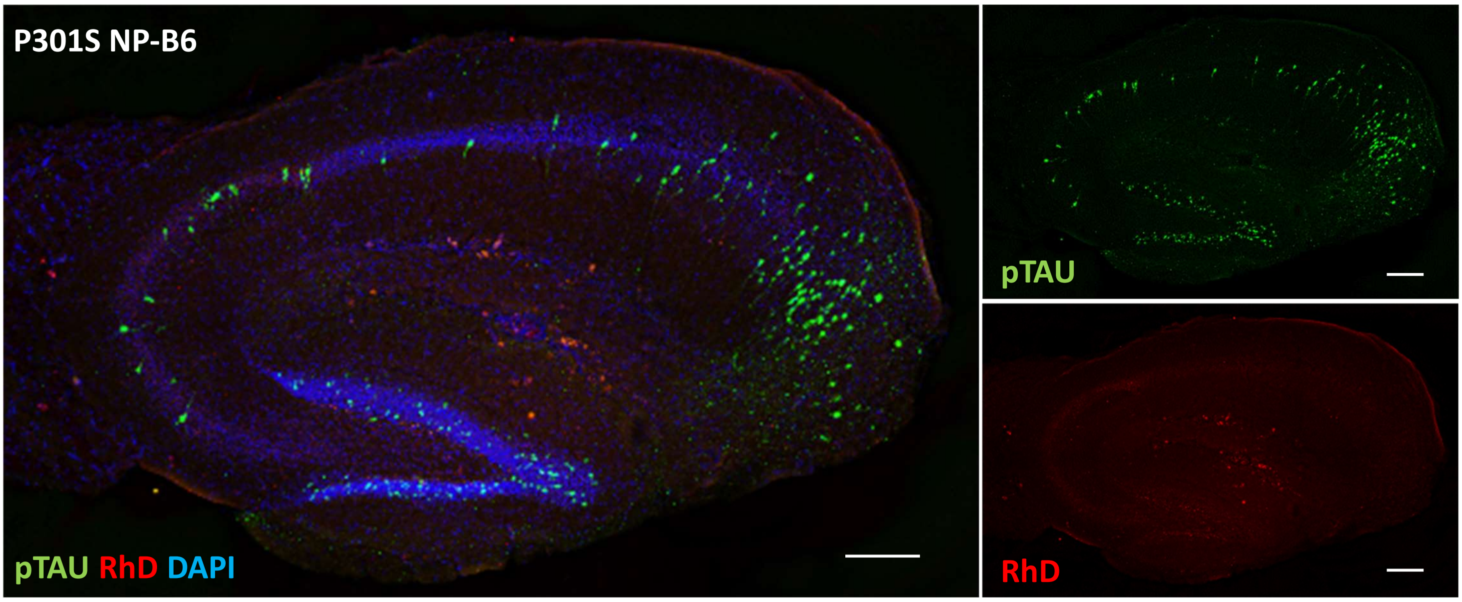

Supplementary Figure S2. Silica-coated rhodamine (RhD) tagged nanoparticle conjugated with B6 antibody (NP-B6, red) does not co-localize with pTAU positive neurons (green) in P301S mouse slices. Nuclei are shown in blue (DAPI). Scalebars 200μm.

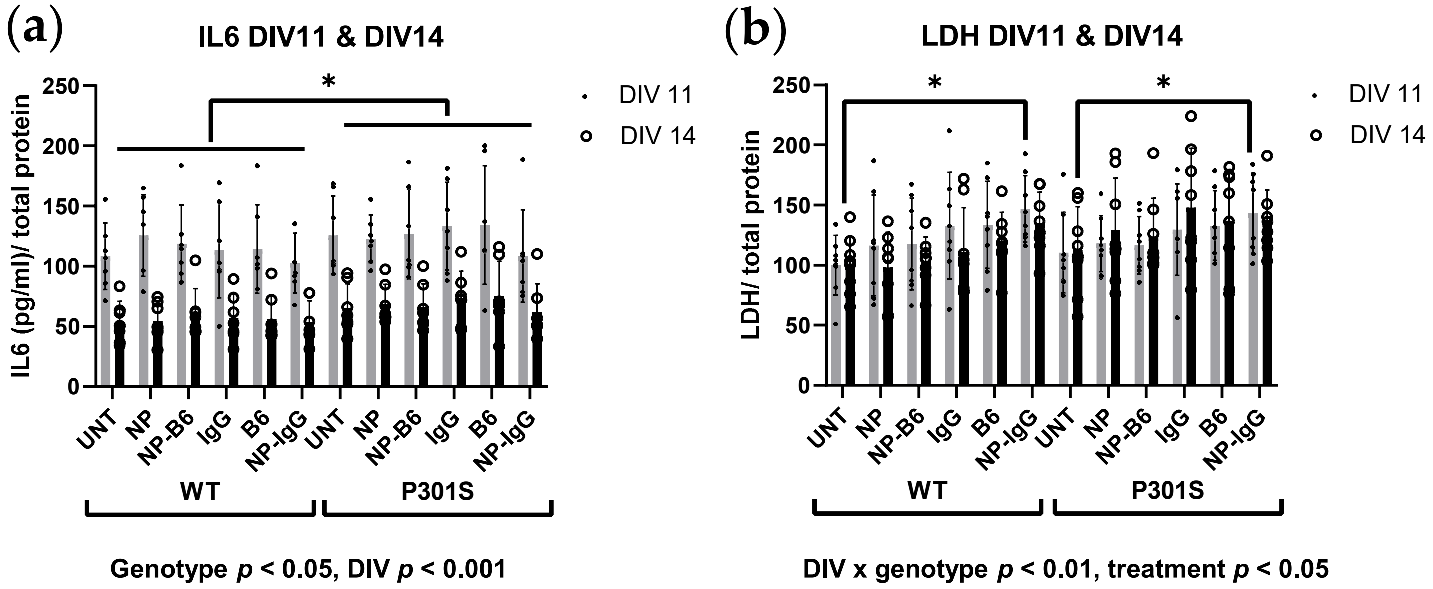

Supplementary Figure S3. P301S slice medium IL6 and LDH levels change during culture in different pattern as compared to wildtypes (WT). (**a**) IL6 levels in relation to total protein content at DIV11 and DIV14. IL6 levels decreased during culture and P301S levels were higher at both timepoints. (**b**) LDH levels at DIV11 and DIV14. In WT medium LDH levels decreased from DIV11 to DIV14 whereas in P301S medium LDH levels increases. Mean ± SD, individual data points correspond to samples from individual wells, n = 7-8. ANOVArm, *$p < 0.05$, Bonferroni. Key: UNT = Untreated, NP = Nanoparticle, NP-B6 = nanoparticle-B6 antibody conjugate, IgG = immunoglobulin G, B6 = anti pTAU antibody, NP-IgG = nanoparticle-immunoglobulin G conjugate, P301S = transgenic hTAU.P301S mice slices, DIV = days *in vitro*.

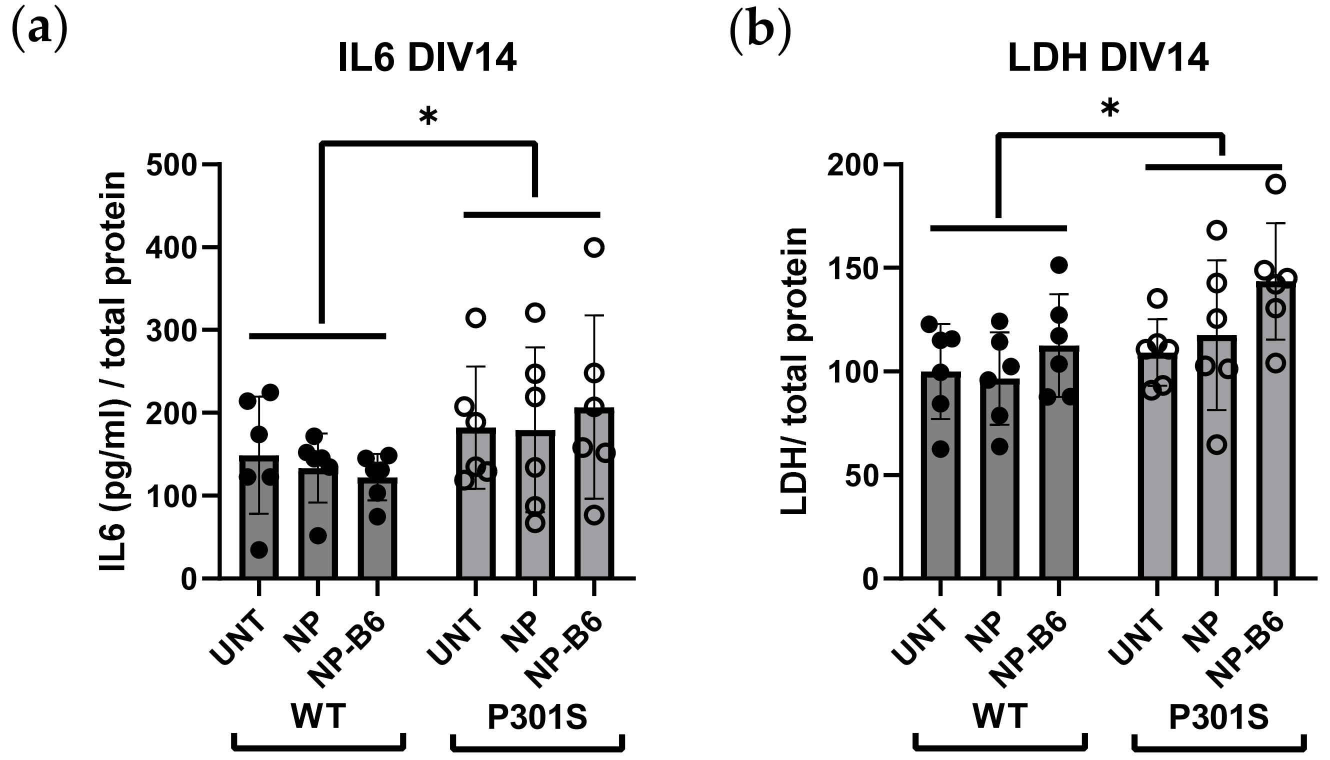

Supplementary Figure S4. P301S slice medium IL6 and LDH levels increased compared to wildtypes (WT) also in experiment 1. (**a**) IL6 levels in relation to total protein content at DIV14. (**b**) LDH levels at DIV14. Mean ± SD, individual data points correspond to samples from individual wells, n = 6. * $p < 0.05$, two-way ANOVA. Key: UNT = Untreated, NP = Nanoparticle, NP-B6 = nanoparticle-B6 antibody conjugate, P301S = transgenic hTAU.P301S mice slices, DIV = days *in vitro.*

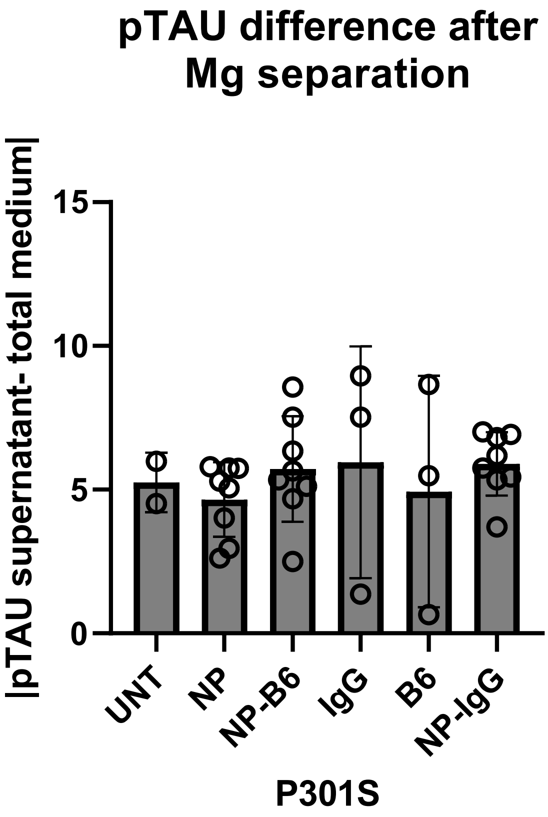

Supplementary Figure S5. Extracellular target engagement to pTAU remains inconclusive for both nanomaterial- B6 conjugates tested in slice culture setting. Difference in pTAU levels between total medium and supernatant in experiment 2. Mean ± SD, individual data points correspond to samples from individual wells, n = 2-8. Key: UNT = Untreated, NP = Nanoparticle, NP-B6 = nanoparticle-B6 antibody conjugate, IgG = immunoglobulin G, B6 = anti pTAU antibody, Mg = magnetic, P301S = transgenic hTAU.P301S mice slices.
